# PARP1 enhances inflammatory cytokine expression by alteration of promoter chromatin structure in microglia

**DOI:** 10.1002/brb3.239

**Published:** 2014-06-09

**Authors:** Ricardo Iván Martínez-Zamudio, Hyo Chol Ha

**Affiliations:** Department of Biochemistry and Molecular & Cellular Biology337 Basic Science Building, 3900 Reservoir Road, Washington, District of Columbia, 20057

**Keywords:** ADP-ribosylation, nucleosome, inflammation, gene expression, cytokine

## Abstract

**Background:**

Poly(ADP-ribose) polymerase 1 (PARP1) is a chromatin-associated enzyme that participates in processes such as transcription and DNA repair through the regulation of chromatin structure. Accumulating evidence suggests an important role for PARP1 enzymatic activity in promoting CNS inflammation by facilitating the expression of inflammatory cytokines in glial cells. However, the molecular mechanisms by which PARP1 enzymatic activity mediates this process are not well understood. In this report we sought to determine the molecular mechanisms by which PARP1 enzymatic activity facilitates the expression of Il1*β* and TNF in LPS-stimulated BV2 cells.

**Methods:**

PARP1 enzymatic activity and histone ADP-ribosylation were measured in LPS-stimulated BV2 cells by radioactive labelling with ^32^P-NAD^+^. To assess the effect of histone ADP-ribosylation on nucleosome structure, in vitro nucleosome remodeling, nuclease accessibility and binding assays were performed. These studies were complemented by chromatin immunoprecipitation assays in resting and LPS-stimulated BV2 cells in order to determine the occupancy of PARP1, nucleosomes and the RelA subunit of NF-*κ*B, as well as ADP-ribosylation, at the Il1*β* and Tnf promoters. Finally, we determined the effect of pharmacological inhibition of PARP1 enzymatic activity on the LPS stimulation-dependent induction of Il1*β* and Tnf mRNA.

**Results:**

Our results indicate that LPS stimulation induces PARP1 enzymatic activity and histone ADP-ribosylation in the chromatin compartment of BV2 cells. In vitro studies show that nucleosome-bound PARP1 disrupts nucleosome structure histone ADP-ribosylation, increasing the accessibility of nucleosomal DNA. Consistent with this PARP1 is constitutively associated with at the Il1*β* and Tnf promoters in resting BV2 cells. Upon stimulation with LPS, ADP-ribosylation is observed at these promoters, and this is correlated with increased recruitment of the transcription factor NF-*κ*B, resulting in robust transcription of these inflammatory cytokines. Accordingly, pharmacological inhibition of PARP1 enzymatic activity reduces NF-*κ*B recruitment, and Il1*β* and Tnf expression in LPS-stimulated microglia.

**Conclusions:**

Collectively, our data suggest that PARP1 facilitates inflammatory cytokine expression in microglia by increasing the accessibility of promoter DNA via histone ADP-riboyslation.

## Introduction

Poly(ADP-ribose) polymerase 1 (PARP1), the founding member of the PARP superfamily, is an abundant chromatin-associated enzyme (D'Amours et al. 1999). Upon recognition of DNA strand breaks, PARP1 catalyzes the synthesis of poly(ADP-ribose) (pADPr) using NAD^+^ as a substrate (Jagtap and Szabo 2005). pADPr is covalently linked to PARP1 and other nuclear targets, including histones, and acts to transiently modify functional properties of the target proteins (Jagtap and Szabo 2005; Gibson and Kraus 2012). Accumulating evidence implicates PARP1 and its enzymatic activity in inflammatory conditions of the central nervous system (CNS) including stroke (reviewed in (Chiarugi 2005)), multiple sclerosis (Chiarugi 2002; Diestel et al. 2003; Farez et al. 2009), and infection (Koedel et al. 2002). For instance, deletion of the *Parp1* gene or pharmacological inhibition of PARP enzymatic activity significantly reduces neuronal cell death and infarct size in animal (Eliasson et al. 1997; Endres et al. 1997) and cell culture (Mandir et al. 2000; Ullrich et al. 2001) models of cerebral ischemia. Similarly, inhibition of PARP enzymatic activity reduces neutrophil infiltration (Chiarugi 2002) and suppresses axonal loss (Diestel et al. 2003; Farez et al. 2009) in mice undergoing experimental autoimmune encephalomyelitis (EAE). Further, in experimental pneumococcal meningitis, both *Parp1*-deficient mice and mice treated with PARP inhibitors display reduced blood brain barrier breaching and meningeal inflammation (Koedel et al. 2002).

Although DNA damage-mediated activation of PARP1 activity in *neurons* in part underlies the pathology of these inflammatory conditions (Eliasson et al. 1997; Endres et al. 1998; Lee et al. 2000), emerging evidence strongly implicates PARP1-regulated inflammatory gene expression in *glial* cells as an important contributor to CNS inflammation. Indeed, primary mixed glial cultures from *Parp1*-deficient mice display reduced *Il1β*, *Il6*, *Tnf,* and *Nos2* expression upon stimulation with lipopolysaccharide (LPS) (Ha et al. 2002; Nakajima et al. 2004), in part due to the impaired activation of transcription factors (TFs) (Ha 2004). Similarly, *Parp1* gene deletion and a PARP inhibitor reduce *Il1β*, *Il6,* and *Tnf* expression in the brain of mice infected with *S. pneumoniae*, and this is correlated with reduced meningitis-induced inflammation (Koedel et al. 2002). Further, antisense RNAi-mediated downregulation of PARP1 and inhibition of PARP enzymatic activity drastically reduce the expression of *Cd11a* in microglial cells and their recruitment to the site of NMDA-mediated neuronal injury in co-culture studies (Ullrich et al. 2001).

Overall, the studies mentioned above suggest an important role for PARP1 enzymatic activity in facilitating inflammatory gene expression upon induction of CNS stress. However, the molecular mechanisms by which PARP1 enzymatic activity mediates this process are not fully understood. Recently, we demonstrated that histone ADP-ribosylation by PARP1 facilitates inflammatory cytokine expression in macrophages by increasing the accessibility of promoter DNA to the master inflammatory TF NF-*κ*B (Martinez-Zamudio and Ha 2012). In this report, we suggest that PARP1 also regulates *Il1β* and *Tnf* expression in BV2 microglial cells through the ADP-ribosylation of nucleosomal histones. In vitro nucleosome remodeling assays demonstrate that PARP1 strongly binds with nucleosomes and destabilizes their structure through histone ADP-ribosylation, which in turn increases the accessibility of nucleosomal DNA. Consistent with this, chromatin immunoprecipitation (ChIP) studies show that PARP1 is constitutively associated with the nucleosome-occupied promoters of *Il1β* and *Tnf* in unstimulated BV2 microglial cells. Upon stimulation with LPS, ADP-ribosylation facilitates NF-*κ*B recruitment to its cognate sites on the *Il1β* and *Tnf* promoters. Accordingly, pharmacological inhibition of PARP1 enzymatic activity reduces NF-*κ*B recruitment to these promoters and the expression of these cytokines in LPS-stimulated microglial cells. Together, these data provide insight into the molecular mechanisms by which PARP1 facilitates cytokine expression in microglia.

## Materials and methods

### Antibodies and reagents

Anti-PARP1 (H250), anti-NF-*κ*B p65 (C20) and protein A/G beads were purchased from Santa Cruz Biotechnologies (Dallas, TX). Anti-histone H3 (1791) was purchased from Abcam (Cambridge, U.K.). Streptavidin agarose was purchased from Millipore (Billerica, MA). LPS from *Salmonella enterica* serotype Typhimurium, micrococcal nuclease (MNase) from *Staphylococcus aureus* were from Sigma (St. Louis, MO). PJ34 was from Alexis Biochemicals (Farmindale, NY). ADP-HPD was from Calbiochem (Billerica, MA). The protease inhibitor cocktail was purchased from Roche (Basel, Switzerland).

### Cell culture

BV2 microglial cells were cultured in Dulbecco's modified Eagle medium (DMEM) supplemented with 10% fetal bovine serum and 0.1 U/ml penicillin-0.1 and *μ*g/mL streptomycin at 37°C. For stimulation of BV2 cells, LPS was used at a final concentration of 1 *μ*g/mL throughout. For pharmacological inhibition of PARP1 enzymatic activity, BV2 cells were pretreated with PJ34 at final concentration of 20 *μ*mol/L 1 h prior to stimulation with LPS.

### PARP enzymatic activity assay

The assay was performed as previously described (Ha et al. 2002). Briefly, BV2 cells were stimulated with LPS for the indicated times and harvested by centrifugation (800 *g*, 3 min, 4°C). The pellet was resuspended in PARP assay buffer (50 mmol/L Tris-HCl [pH 8.0], 28 mmol/L KCl, 10 mmol/L MgCl_2_, 0.01% digitonin, 1 mmol/L dithiothreitol, and ^32^P-NAD^+^ [0.8 *μ*Ci/nmol, 0.5 *μ*Ci/10^6^ cells]) and incubated on ice for 20 min. The reaction was quenched by addition of the PARP and poly(ADP-ribose glycohydrolase (PARG) inhibitors PJ34 (10 *μ*mol/L) and ADP-HDP (500 nmol/L). The cells were subsequently lysed as described below. Equal amounts of protein were separated in 12% NuPAGE gels. PARP enzymatic activity was visualized by autoradiography.

### Protein extraction

Protein was extracted with nonionic detergent as previously described (Martinez-Zamudio and Ha 2012).

### Immunofluorescence microscopy

Cells were seeded on sterile glass coverslips in 12-well plates 1 day prior to experimentation. Control, LPS-stimulated and LPS-stimulated BV2 microglial pretreated with PJ34 were fixed in PBS containing 3.7% paraformaldehyde for 30 min at room temperature. BV cells were washed and subsequently permeabilized in PBS containing 2% BSA and 0.3% Triton X-100 for 30 min at room temperature. Staining of p65 was performed overnight at 4°C at a 1:100 dilution. Coverslips were washed three times for 10 min at room temperature with PBS and subsequently incubated with a rhodamine-conjugated anti-rabbit goat antibody for 1 h at 1:2000 dilution. Coverslips were washed three times for 10 min at room temperature, dried, and subsequently mounted on slides containing Vectashield. Staining was visualized in a fluorescent microscope and images were processed in Image J. Nuclear localization was determined by counting 100 cells from random fields using DAPI as a reference.

### Sucrose gradient analysis of MNase-digested chromatin

LPS-stimulated BV2 cells were subjected to the PARP enzymatic activity assay and immediately fixed with 0.5% formaldehyde. Subsequently, cells were fractionated as previously described (Wysocka et al. 2001), and the nuclear fraction was digested with 40 U MNase for 10 min at 37°C. The MNase-solubilized fraction was collected by centrifugation (1700 *g*, 5 min, 4°C) and carefully layered onto 10–40% sucrose gradients prepared in Tris-EDTA (TE) buffer containing 150 mmol/L NaCl. Centrifugation was performed at 137,000 *g* for 16 h at 4°C. Fractions were collected from the bottom of the tube, and equal volumes were analyzed by SDS-PAGE, autoradiography and immunoblot.

### Real-time reverse-transcription polymerase chain reaction (qRT-PCR)

RNA extraction and RNA expression by quantitative RT-PCR were performed as previously described (Martinez-Zamudio and Ha 2012).

### Chromatin immunoprecipitation

Control and LPS-stimulated BV2 microglial cells in the presence or absence of pretreatment with PJ34 were cross-linked with 0.5% formaldehyde in PBS for 8 min at room temperature. The reaction was quenched by addition of 125 mmol/L glycine for 8 min at room temperature, and the cells were collected by centrifugation (800 *g*, 3 min, 4°C). The cells were subjected to biochemical fractionation (Wysocka et al. 2001), and the chromatin fraction was resuspended in SDS lysis buffer (1% SDS, 20 mmol/L Tris-HCl pH 8.0, and 2 mmol/L EDTA) and sonicated with a Branson sonicator model 250 equipped with a tip model 102C (40% output, 4 times for 10 s). Soluble chromatin was obtained by centrifugation (12,000 *g* for 15 min at 4°C). The average size of the chromatin fragments were 200–700 bp. Sonicated chromatin was diluted 10-fold in chromatin immunoprecipitation (ChIP) dilution buffer (1.1% Triton X-100, 0.1% SDS, 167 mmol/L NaCl, 22.3 mmol/L Tris-HCl [pH 8.0], and 2.2 mmol/L EDTA) and precleared with 30 *μ*L of a 50% protein A/G agarose slurry for 2 h at 4°C. Immunoprecipitation was performed using anti-PARP-1, anti-NF-*κ*B p65, and anti-histone H3 antibodies (2 *μ*g each). Isotype-matched IgG and a mock precipitation without antibodies were used as negative controls for immunoprecipitation. The immune complexes were isolated by incubation with protein A/G beads overnight at 4°C. The beads were collected by centrifugation and washed 3 times with a wash buffer containing 1% Triton X-100, 0.1% SDS, 150 mmol/L NaCl, 20 mmol/L Tris-HCl (pH 8.0), and 2 mmol/L EDTA, once with wash buffer containing 300 mmol/L NaCl, and twice with TE buffer (pH 8.0). Immune complexes were eluted twice with an elution buffer containing 1% SDS and 100 mmol/L sodium acetate (NaOAc) at 37°C for 30 min. After reversal of cross-links (4 h, 65°C), the samples were treated with proteinase K and RNase A (500 *μ*g/mL each, 1 h, 42°C), and DNA was purified by phenol–chloroform extraction. DNA was precipitated with ethanol (EtOH) and resuspended in TE. One-twentieth of the recovered DNA was used for PCRs (p65, 32 cycles; H3, 26 cycles; PARP1, 28 cycles; biotin-NAD^+^, 29 cycles). For biotinylated-NAD^+^ ChIP experiments, the procedure was carried out identically except that cells were labeled with 50 *μ*mol/L biotinylated NAD^+^. ADP-ribosylated complexes were isolated with streptavidin-coupled agarose beads. Input DNA and PCR products were analyzed in 1.5% Tris borate-EDTA (TBE) agarose gels and were visualized by SYBR green staining. Primers are available upon request.

### Mononucleosome assembly

Mononucleosomes were assembled by the ‘salt jump’ method (Hamiche et al. 2001). Briefly, the 601 nucleosome positioning sequence (Lowary and Widom 1998) and a 156 fragment from the *Il1β* promoter (−135 to +21 relative to the transcription start site) were amplified by PCR and gel purified using the QIAGEN gel extraction kit. Purified fragments (3 *μ*g) were incubated with chicken core histones at a weight ratio of 1:1.4 (DNA to histones) in 100 *μ*L reactions containing 1 mg/mL BSA and 2mol/L NaCl for 3 h at 37° C. A buffer containing 20 mmol/L Tris-HCl (pH 7.5), 100 mg/mL BSA, 1 mmol/L EDTA, 0.5 mmol/L PMSF, 5 mmol/L DTT was added drop-wise in volumes of 100, 200, 400 and 600 *μ*L, with 15 min incubations at 37°C after each addition. Assembled nucleosomes were assessed by native agarose gel electrophoresis and SYBR green staining of DNA.

### Generation of Poly(ADP-ribosyl)ated-PARP1 (pADPr-PARP1)

One microgram recombinant PARP1 was incubated with 0.2, 2, 20 and 75 *μ*mol/L NAD^+^, and 2.5 *μ*mol/L of a single-stranded oligonucleotide in the presence of 1.5 mmol/L MgCl_2_. The reactions were allowed to proceed for 20 min at room temperature and subsequently quenched by addition of 1 mmol/L PJ34. pADPr-PARP1 was isolated by centrifugation on a Microcon concentrator (10 kDa cut-off; Millipore) according to the manufacturer's instructions. pADPr-PARP1 was diluted to a concentration of 100 ng/*μ*L and stored at −20°C for subsequent use.

### Nucleosome remodeling assay

The PARP1 enzymatic activity-dependent nucleosome remodeling assay was carried out identically as described in (Martinez-Zamudio and Ha 2012).

### NF-*κ*B nucleosome-binding assay

Nucleosomes reconstituted with the *Il1β* proximal promoter sequence were remodeled with PARP1 and NAD^+^ (0, 2, 5, 10 and 20 *μ*mol/L). Subsequently, p65/RelA was added and the reactions mixtures were incubated for 20 min at room temperature. Reactions were quenched on ice for 5 min and analyzed by electrophoretic mobility shift assay (EMSA). Results were visualized by SYBR green staining of DNA. The molar ratio of p65 to nucleosomes was 4–1.

### DNase I hypersensitivity

DNase hypersensitivity assays were performed exactly as described in (Martinez-Zamudio and Ha 2012) using 601 nucleosomes ^32^P-labeled at the 5′ end of the antisense strand.

## Results

### LPS stimulation leads to histone ADP-ribosylation by PARP1 in BV2 cells

**Figure 1:**
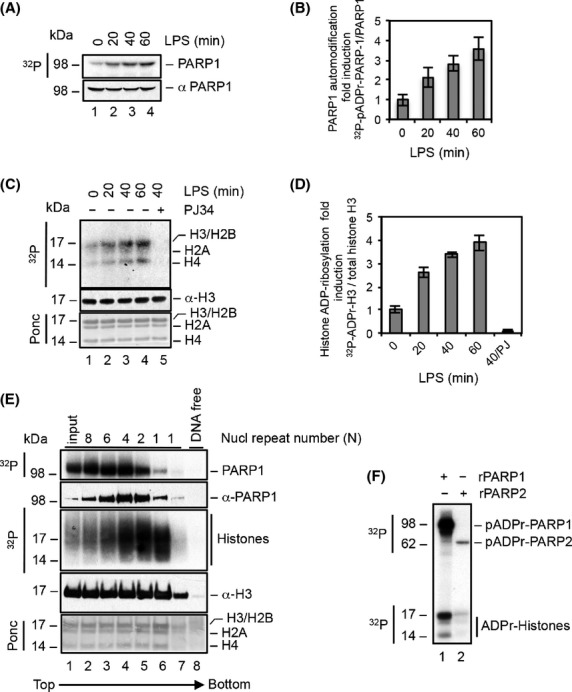
LPS stimulation leads to PARP1 enzymatic activation and histone ADP-ribosylation in microglia. (A) BV2 microglial cells were stimulated with LPS for the indicated times and subsequently subjected to the PARP enzymatic activity assay. Protein was extracted with nonionic detergent and analyzed by NuPAGE, autoradiography and immunoblot (A, C and E). (B) Graphical representation of three experiments as in A. Bars indicate SEM (C) BV2 microglial cells were stimulated with LPS for the indicated times and subjected to the PARP enzymatic activity assay. Histones were isolated by acid extraction of nuclei and analyzed as in A. PJ34 (10 *μ*mol/L) was added 1 h prior to stimulation (lane 5). (D) Graphical representation of three experiments as in C. Bars indicate SEM (E) LPS-stimulated BV2 microglia were subjected to biochemical fractionation and the chromatin fraction was digested with MNase. Digested chromatin was layered on a 10–40% sucrose gradient and centrifuged overnight at 137,000 *g*. Equal volumes of each fraction were analyzed. (F) rPARP1 and rPARP2 were incubated in the presence of assembled nucleosomes at a molar ratio of 2:1 in the presence of 100 nM ^32^P-NAD^+^. Reactions were analyzed by NuPAGE and autoradiography.

To better understand the role of PARP1 enzymatic activity in inflammatory gene expression of the CNS, we characterized PARP1 enzymatic activity in BV2 microglial cells. BV2 cells were stimulated with LPS for the indicated times and then subjected to the PARP enzymatic activity assay. Stimulation with LPS consistently resulted in a time-dependent induction of PARP1 enzymatic activity, as revealed by increased levels of poly(ADP)ribosylated-PARP1 (pADPr-PARP1) (Fig. 1A and B). PARP1 is a chromatin-associated enzyme that, in addition to catalyzing its auto-modification, also attaches pADPr chains to other substrates such as histones (Jagtap and Szabo 2005). We recently showed that stimulation of macrophages with LPS enhances histone ADP-ribosylation (Martinez-Zamudio and Ha 2012). To assess whether histone ADP-ribosylation occurred in microglia, we analyzed acid-extracted histones from LPS-stimulated BV2 cells. Consistent with studies of PARP1 enzymatic activity, LPS stimulation significantly increased the level of ADP-ribosylated histones in a time-dependent manner (Fig. 1C and D). Importantly, histone ADP-ribosylation was dependent on PARP1 enzymatic activity, as pretreatment of BV2 cells with the PARP inhibitor PJ34 completely abrogated this process (Fig. 1C lane 5, and D). To evaluate whether histone ADP-ribosylation by PARP1 occurred at the chromatin level, density gradient analyses of micrococcal nuclease (MNase)-digested chromatin from LPS-stimulated BV2 cells were performed. Both pADPr-PARP1 and ADP-ribosylated histones were observed in all the nucleosome-containing fractions of the gradient (Fig. 1E, lanes 2–7). In contrast, pADPr-PARP1 and ADP-ribosylated histones were not detected in the DNA-free fraction (Fig. 1E, lane 8). Thus, PARP1 was associated with nucleosomes in the chromatin compartment of BV2 cells. Furthermore, PARP1 was the major enzyme catalyzing the modification of histones in microglia, as no PARP2 enzymatic catalytic activity was detected in cells (data not shown) and recombinant PARP2 (rPARP2) showed minimal catalytic activity toward assembled nucleosomes in vitro as compared to rPARP1 (Fig. 1F). Collectively, these results indicate that stimulation of BV2 microglial cells with LPS induces PARP1 enzymatic activity and the ADP-ribosylation of nucleosomal histones.

### PARP1-mediated histone ADP-ribosylation disrupts nucleosome structure

**Figure 2:**
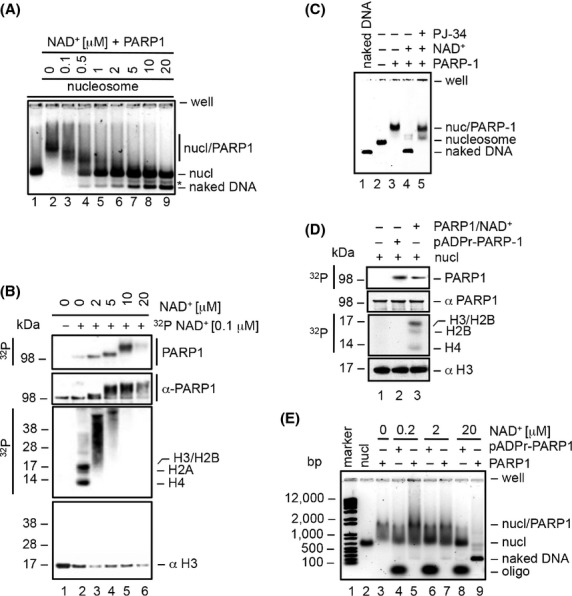
Histone ADP-ribosylation destabilizes nucleosome structure. (A) PARP1 was incubated with the 601 nucleosomes and the indicated NAD^+^ concentrations for 20 min at room temperature. After quenching by addition of 1 mmol/L PJ34, reactions were analyzed by native agarose gel electrophoresis and visualized by SYBR Green staining of DNA (A, C and E). (B) An identical set of reactions as in A was supplemented with 100 nM ^32^P-NAD^+^. Reactions were analyzed by NuPAGE, autoradiography and immunoblot. The decrease in immunoreactivity of histone H3 likely reflects the interference of ADPr with the epitope recognized by the antibody. (C) PARP1 was incubated with 20 *μ*mol/L NAD^+^ with or without pretreatment with 100 *μ*mol/L PJ34 (lanes 4 and 5). Reactions were performed exactly as in A. (D) PARP1 and 100 nM ^32^P-NAD^+^ or ^32^P-pADPr-PARP1 was incubated with the 601 nucleosomes for 20 min at room temperature. Reactions were quenched by boiling and subsequently analyzed by NuPAGE, autoradiography and immunoblot. (E) PARP1 or pADPr-PARP1 was incubated with the 601 nucleosomes and the indicated NAD^+^ concentrations for 20 min at room temperature.

The limited accessibility of DNA in the nucleosome represents an obstacle to most transcription factors and components of the transcriptional machinery (Workman and Kingston 1998; Becker and Horz 2002). As such, the alteration of nucleosome structure is in some cases critical to facilitate transcription (Mizuguchi et al. 2001; Wysocka et al. 2006). The covalent attachment of the highly negatively charged ADP-ribose polymer to histones is likely to alter nucleosome structure (Boulikas 1991), which in turn could facilitate gene transcription (Martinez-Zamudio and Ha 2012; Petesch and Lis 2012). We performed a series of in vitro experiments to carefully assess the effect of PARP1-mediated histone ADP-ribosylation on nucleosome structure. Nucleosomes reconstituted with the 601 nucleosome positioning sequence (NPS) (Lowary and Widom 1998) were incubated with PARP1 in the presence of various NAD^+^ concentrations and subsequently analyzed by electrophoretic mobility shift assay (EMSA). Addition of PARP1 to the nucleosomes resulted in a retarded migration, indicating that PARP1 stably bound to the nucleosomes (Fig. 2A, lane 2). Co-addition of NAD^+^ resulted in dissociation of PARP1 from nucleosomes and generation of naked DNA in a manner dependent on the NAD^+^ concentration (Fig. 2A, lanes 3–9). Importantly, these processes correlated with the extent of both PARP1 and histone ADP-ribosylation (Fig. 2B), and were dependent on the enzymatic activity of PARP1 (Fig. 2C). These results indicate that PARP1 enzymatic activity promotes nucleosome destabilization. PARP1 is known to be the major target of ADP-ribosylation in the cell (D'Amours et al. 1999), and auto-modified PARP1 has been generally thought to disrupt nucleosome structure (Realini and Althaus 1992; Tulin and Spradling 2003). To assess whether auto-modified PARP1 or histone ADP-ribosylation is responsible for the generation of naked DNA, the 601 nucleosomes were incubated with purified auto-modified PARP1 or PARP1 and NAD^+^ (Fig. 2E). pADPr-PARP1 bound to nucleosomes and eventually dissociated from nucleosomes, without generating naked DNA, in a manner dependent on NAD^+^ concentration and therefore the extent of auto-modification (Fig. 2E, lanes 4, 6, and 8). Importantly, pADPr-PARP1 was unable to ADP-ribosylate nucleosomal histones under these conditions (Fig. 2D), demonstrating the purity of the pADPr-PARP1 preparation. In contrast, generation of naked DNA was only observed upon incubation with PARP1 and NAD^+^ (Fig. 2E, lanes 5, 7, and 9). Collectively, these results indicate that histone ADP-ribosylation was responsible for disrupting nucleosome structure, whereas highly modified PARP1 was unable to associate with nucleosomes.

### PARP1 enzymatic activity increases the accessibility of nucleosomal DNA and facilitates cytokine transcription by enhancing NF-*κ*B recruitment to the promoters of genes in LPS-stimulated microglia

Interleukin 1 beta (*Il1β*) and Tumor Necrosis Factor (*Tnf*) are primary response cytokines that contribute to the pathology of various inflammatory conditions of the CNS (Bauer et al. 1993; Chiarugi 2002; Allan et al. 2005; Vezzani et al. 2011) by directly activating microglia and astrocytes (Proescholdt et al. 2002; Depino et al. 2005), enhancing recruitment of leukocytes (Ferrari et al. 2004; Shaftel et al. 2007), and facilitating the generation of reactive oxygen and nitrogen species (Chao et al. 1996). In addition, pharmacological inhibition of PARP1 enzymatic activity and PARP1 deficiency reduces inflammation-induced *Il1β* and *Tnf* expression in mixed glial cultures (Ha et al. 2002; Nakajima et al. 2004). Furthermore, increasing evidence demonstrates that PARP1 is constitutively associated with the promoters of genes in a variety of cell types (Ju et al. 2004; Krishnakumar et al. 2008; Martinez-Zamudio and Ha 2012), suggesting a potential epigenetic link between ADP-ribosylation and gene regulation.

**Figure 3:**
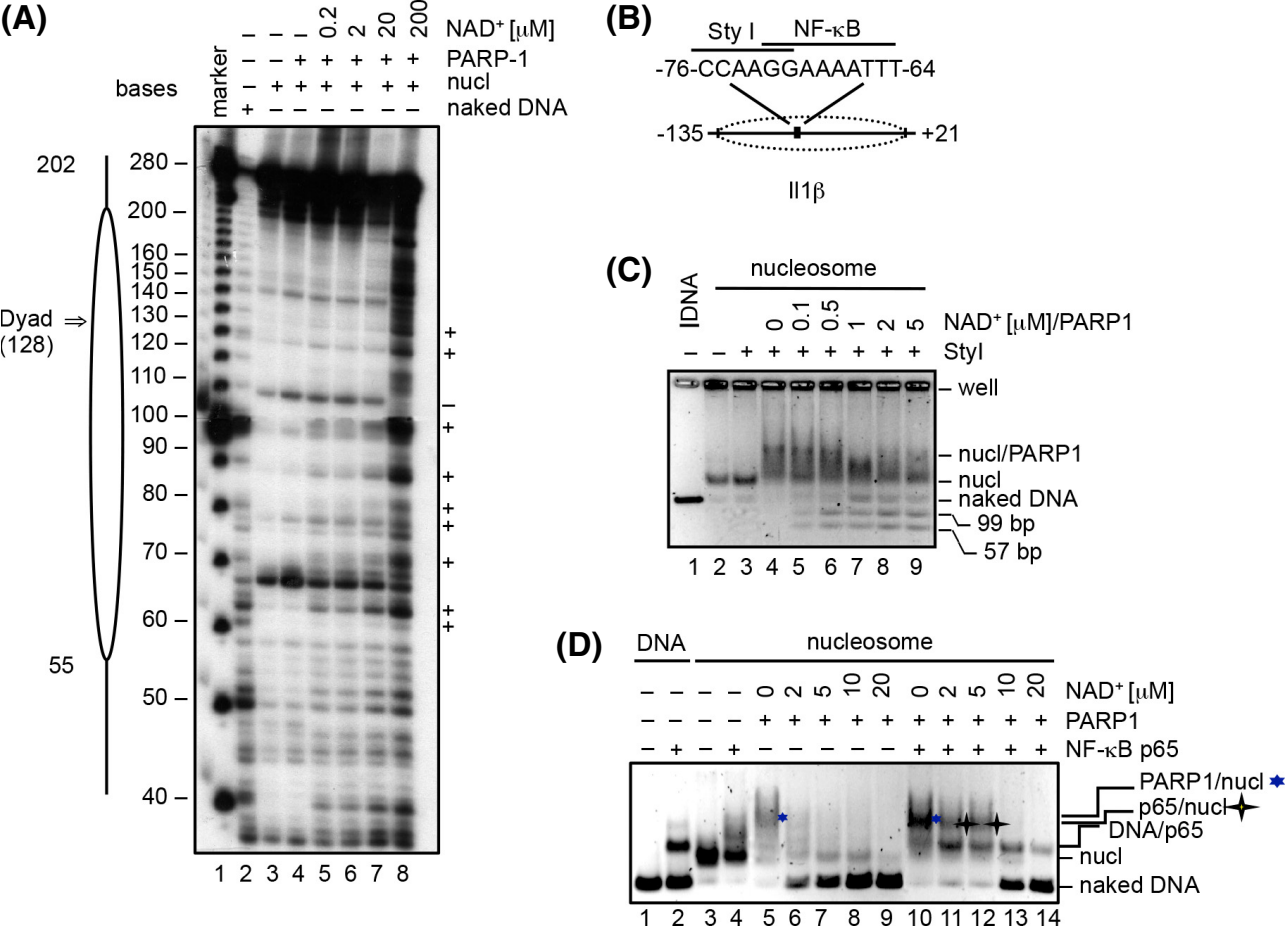
Histone ADP-ribosylation by PARP1 increases accessibility of nucleosomal DNA. (A) 601 nucleosomes labeled with ^32^P-NAD^+^ at the 5′ end of the antisense strand were incubated with PARP1 and the indicated NAD^+^ concentrations. Reactions were subsequently incubated with DNAse I and analyzed in a denaturing 8% polyacrylamide gel followed by autoradiography. +, DNase I-hypersensitive sites. Lane 1, DNA marker; lane 2, digested DNA control; lane 3, digested nucleosomes. The trace 10 bp repeating pattern observed in lane 2 is due to contamination from lane 1. (B) Schematic representation of the nucleosomes reconstituted with the *Il1β* proximal promoter indicating the NF-*κ*B binding site and the Sty I restriction site. (C) The *Il1β* nucleosomes were incubated with PARP1 and the indicated NAD^+^ concentrations for 20 min at room temperature. Subsequently, 20 units of StyI were added and reactions were incubated at 37°C for 1 h. Reactions were stopped by addition of EDTA and heat inactivation at 65°C for 10 min. Reactions were analyzed by native agarose gel electrophoresis and DNA visualized by SYBR Green staining (B and C). (D) The *Il1β* nucleosomes were remodeled with the indicated NAD^+^ concentrations and subsequently incubated with or without p65 for 20 min at room temperature. The blue asterisk indicates the PARP1/nucleosome complex. The yellow star indicates a putative p65/nucleosome complex.

To further understand the role of PARP1 enzymatic in facilitating cytokine expression via the regulation of nucleosome structure, we performed DNAse I hypersensitivity assays with nucleosomes reconstituted with the 601 positioning sequence. The 601 nucleosomes were end-labeled on the antisense strand with ^32^P and subsequently digested with DNAse I. Under these conditions, the expected 10-11 bp DNA helical digestion pattern (Vitolo et al. 2001) was observed (Fig. 3A, lane 3). Addition to the nucleosomes of either PARP1 (Fig. 3A, lane 4) or NAD^+^ alone (Fig. S1) did not alter the digestion pattern. However, upon co-addition of NAD^+^, the digestion pattern significantly changed, resulting in increased digestion particularly in the core particle region (Fig. 3A, lanes 5–8). Of note, the increase in DNAse I sensitivity correlated with NAD^+^ concentration, and therefore with the extent of histone ADP-ribosylation. These studies suggest that PARP1-catalyzed histone ADP-ribosylation likely alters nucleosome structure by weakening the interaction between histones and DNA, leading to increased accessibility of nucleosomal DNA.

We then performed restriction enzyme accessibility (REA) and EMSA assays on nucleosomes reconstituted with the *Il1β* proximal promoter sequence. Importantly, a StyI site and an NF-*κ*B consensus element present in *Il1β* proximal promoter sequence localize near the dyad axis in the assembled nucleosomes (Fig. 3B). Incubation of the *Il1β* nucleosomes with StyI did not result in the generation of the expected DNA cleavage products (Fig. 3C, lane 3), indicating that the accessibility of the StyI restriction site is limited in the context of the nucleosome. Similarly, addition of PARP1 to the reaction mixture did not increase the accessibility of the StyI restriction site (Fig. 3C, lane 4). In contrast, addition of PARP1 and NAD^+^ led to an NAD^+^ concentration-dependent increase in the generation of the expected cleavage fragments (Fig. 3C, lanes 5–9). This result is reminiscent of the DNAse I hypersensitivity assays and suggests that histone ADP-riboyslation by PARP1 increases accessibility of StyI to its site in the context of the nucleosome. To assess whether histone ADP-ribosylation could facilitate binding of the master inflammatory TF NF-*κ*B to its consensus site in the *Il1β* nucleosomes, binding assays were performed. Incubation of the RelA/p65 subunit of NF-*κ*B with the naked *Il1β* promoter fragment resulted in a retarded migration pattern upon electrophoresis, demonstrating that p65 stably associates with its binding site in the *Il1β* promoter (Fig. 3D, lanes 1 and 2). However, p65 was barely able to bind to its cognate site in the reconstituted *Il1β* nucleosomes, indicating that the nucleosome limits the accessibility of DNA to p65 (Fig. 3D, lanes 3 and 4). As observed with the 601 nucleosomes, PARP1 bound to the *Il1β* nucleosomes and promoted their destabilization upon addition of NAD^+^ (Fig. 3D, lanes 5–9). When p65 was incubated with PARP1 and low-NAD^+^ concentrations (2 and 5 *μ*mol/L), the p65/naked *Il1β* promoter complex was observed along with a new band with a slightly faster migration than the PARP1/nucleosome complex (Fig. 3D, lanes 11 and 12). As PARP1 was already dissociated from the nucleosomes at these NAD^+^ concentrations in the absence of p65 (Fig. 3D, lanes 6 and 7), this new band likely represents a p65/nucleosome complex. At higher NAD^+^ concentrations, the histone octamer was completely displaced, resulting in the formation of a p65/DNA complex along with naked DNA (Fig. 3D, lanes 13–14). Collectively, these results indicate that histone ADP-ribosylation increases the accessibility of the *κ*B site in the nucleosome, thus enhancing p65 binding.

**Figure 4:**
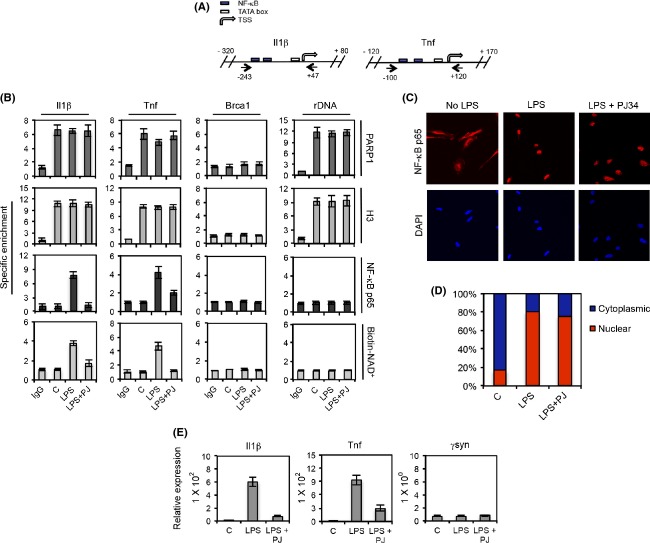
PARP1 enzymatic activity regulates *Il1β* and *Tnf* expression by facilitating NF-*κ*B recruitment at the *Il1β* and *Tnf* promoters. (A) Schematic representation of the *Il1β* and *Tnf* proximal promoters. Primer positions are indicated by the black arrows. NF-*κ*B sites, TATA box and transcription start site are indicated. (B) Semiquantitative ChIP analyses of H3, PARP1, ADP-ribosylation and NF-*κ*B at the promoters of *Il1β*, *Tnf*, *Brca1* and *rDNA* in control and LPS-stimulated (60 min) microglia with or without pretreatment with PJ34. Gel band signals were normalized to a No Ab control, and the ratio of IgG signal to the No Ab signal was set to 1. The average of three independent experiments is presented. Bars, SEM (C). BV2 cells with or without pretreatment with PJ34 were stimulated with LPS for 60 min. The nuclear translocation of NF-*κ*B was monitored by indirect immunofluorescence using specific antibodies (top panel). Nuclear counterstaining is provided by DAPI (lower panel). (D). Quantification of 3 experiments as in C. 100 cells from random fields were counted for p65 nuclear translocation using DAPI as a reference. The nuclear translocation of p65 was statistically significant in LPS-stimulated BV2 cells with or without PJ34 pretreatment relative to stimulated cells (*P* < 0.01 by one-way ANOVA followed by Tukey's HSD test). The SEM was < 15%. (E) Total RNA from control and LPS-stimulated (90 min) microglia with or without pretreatment with PJ34 was extracted, and the expression of *Il1β* and *γsyn* was measured by qRT-PCR. Relative expression was determined as the ratio of mRNA levels of the indicated genes to *β*-actin levels and is relative to expression in unstimulated cells. Bars, SEM Similar results were obtained when cytokine mRNA expression was normalized to *rDNA*. The difference between *Il1β* and *Tnf* expression in LPS-stimulated cells relative to control cells, and LPS-stimulated, PJ34-pretreated BV2 cells relative to LPS-stimulated cells was found to be statistically significant by an ANOVA test followed by Tukey's HSD test (*P* < 0.01). No statistical significance was found for *γsyn* mRNA in all conditions tested.

To provide a cellular counterpart to these findings, we performed ChIP analyses of the *Il1β* and *Tnf* proximal promoters in control and LPS-stimulated BV2 cells (Fig. 4A). We initially determined nucleosome and PARP1 occupancy profiles at the *Il1β* and *Tnf* promoters using antibodies against histone H3 and PARP1. These assays consistently revealed nucleosome and PARP1 occupancy at the *Il1β* and *Tnf* promoters in both resting and LPS-stimulated BV2 cells (Fig. 4B, top 2 panels). Furthermore, the reproducibility of the nucleosome and PARP1 occupancy patterns suggested that these patterns may be epigenetically preserved (Guetg et al. 2012).

As LPS stimulation induced PARP1 enzymatic activity and histone ADP-ribosylation, and histone ADP-ribosylation increased the accessibility of nucleosomal DNA, we investigated whether histone ADP-ribosylation could enhance NF-*κ*B recruitment to these promoters in LPS-stimulated microglial cells. To detect ADP-ribosylation at the promoters of *Il1β* and *Tnf*, we performed the PARP enzymatic assay using biotinylated NAD^+^ to tag ADP-ribosylated proteins (Tulin and Spradling 2003). Subsequently, chromatin was prepared by sonication and ADP-ribosylated proteins were isolated by a pull-down step with streptavidin beads. Using this approach, an LPS stimulation-specific increase in ADP-ribose synthesis was detected at the *Il1β* and *Tnf* promoters (Fig. 4B, bottom panel). This event was mediated by PARP1 enzymatic activity, as pretreatment of BV2 cells with PJ34 abrogated the LPS stimulation-induced ADP-ribose synthesis (Fig. 4B, bottom panel). We then monitored NF-*κ*B recruitment by performing ChIP with antibodies to the p65 subunit of NF-*κ*B. p65 was recruited to the *Il1β* and *Tnf* promoters in an LPS stimulation-specific manner in cells (Fig. 4B, third panel from top). Interestingly, p65 recruitment was greatly reduced in stimulated BV2 cells pretreated with PJ34 (Fig. 4B, third panel from top). The inhibition of p65 recruitment by PJ34 was not due to impaired p65 nuclear translocation, as this process remained unimpaired in PJ34-treated microglia (Fig. 4C and D). Therefore, the ChIP analyses indicated that ADP-ribose synthesis and p65 recruitment at the promoters of *Il1β* and *Tnf* were correlated processes.

To test whether these phenomena were specific to the *Il1β* and *Tnf* promoters, we monitored the nucleosome, PARP1, p65, and ADP-ribose ChIP profiles of the promoters of two genes whose expression is insensitive to LPS stimulation; the low-nucleosome density *Brca1* promoter (Fig. 4B) and the nucleosome and PARP1-occupied *rDNA* promoter (Guetg et al. 2012) (Fig. 4B). As expected, neither p65 recruitment nor ADP-ribosylation was observed at these promoters upon stimulation with LPS (Fig. 4B, bottom panels). In addition, neither LPS stimulation nor pretreatment with PJ34 altered the PARP1 or H3 ChIP patterns at these promoters (Fig. 4B, top panels).

We investigated the biological significance of these findings by measuring *Il1β* and *Tnf* expression in LPS-stimulated BV2 cells. Real-time RT-PCR analysis revealed that both *Il1β* and *Tnf* mRNA were undetectable in resting microglial cells (Fig. 4E). Upon stimulation with LPS, a drastic increase in *Il1β* and *Tnf* mRNA was observed (Fig. 4E). Importantly, when BV2 microglial cells were pretreated with PJ34, the LPS stimulation-induced *Il1β* and *Tnf* mRNA expression was impaired (Fig. 4E), indicating that the expression of these genes is regulated by PARP1 enzymatic activity. In contrast, the expression pattern of an irrelevant control gene, *γsyn*, remained unchanged in all conditions tested (Fig. 4E). Collectively, the nucleosome remodeling, ChIP and mRNA expression studies support the notion that PARP1 facilitates *Il1β* and *Tnf* expression in LPS-stimulated microglia by enhancing NF-*κ*B binding at the promoters of these genes via histone ADP-ribosylation.

## Discussion

Diverse pathological CNS conditions including infection, trauma or neurodegenerative disorders induce an inflammatory response that is mediated by activated microglia, astrocytes, and infiltrating macrophages (Kriz 2006; Jin et al. 2010). These cells release inflammatory cytokines and mediators, which promote neuronal cell death in part by the additional recruitment of immune cells (Schilling et al. 2003; Price et al. 2004). Accumulating evidence suggests an important role for PARP1 in facilitating neurotoxicity via the regulation of inflammatory gene expression in microglia and astrocytes (Chiarugi 2002; Ha et al. 2002; Koedel et al. 2002; Diestel et al. 2003; Farez et al. 2009). Despite this, the molecular mechanism by which PARP1 facilitates this process remains poorly understood. In this report, we presented evidence supporting a chromatin-based mechanism by which PARP1 facilitates *Il1β* and *Tnf* expression in LPS-stimulated microglia. PARP1 was constitutively associated with the nucleosome-occupied promoters of *Il1β* and *Tnf* in resting microglial cells. Upon stimulation with LPS, PARP1 was enzymatically activated and catalyzed the ADP-ribosylation of nucleosomal histones. Histone ADP-ribosylation increased the accessibility of nucleosomal DNA and enhanced the recruitment of NF-*κ*B to the promoters of *Il1β* and *Tnf*, thus facilitating their transcription.

Previous reports have shown that inhibition of PARP1 enzymatic activity or deletion of PARP1 is protective in experimental models of multiple sclerosis (Chiarugi 2002; Diestel et al. 2003; Farez et al. 2009), ischemia-reperfusion (Eliasson et al. 1997; Ullrich et al. 2001) and infection (Ha et al. 2002; Koedel et al. 2002; Ha 2004; Nakajima et al. 2004). Notably, in experimental meningitis, PARP1-deficient mice and mice treated with the PARP inhibitor, 3 aminobenzamide (3-AB), display reduced leukocyte infiltration and meningeal inflammation, and this is correlated with impaired expression of *Il1β*, *Il6* and *Nos2 (Koedel* et al. 2002). Furthermore, in an organotypic culture model of ischemia-reperfusion, pretreatment with 3-AB reduces microglial cell recruitment to the site of injury and associated neurotoxicity via the impaired expression of the integrin CD11A (Ullrich et al. 2001). In addition, antisense RNA downregulation of PARP1, PARP inhibitors, and PARP1-deficiency have all been shown to ameliorate the progression of EAE in mice by reducing expression of *Nos2*, *Tnf,* and *Ccl2* (Chiarugi 2002; Diestel et al. 2003; Farez et al. 2009). Although these studies implicate a role for PARP1 enzymatic activity in inflammatory conditions of the CNS, the targets of PARP1 enzymatic activity have remained poorly characterized.

In this report, we showed that the main targets of PARP1 enzymatic activity in LPS-activated BV2 cells are PARP1 itself and the four core histones. Importantly, both PARP1 and the core histones were ADP-ribosylated at the chromatin compartment, where the modification of nucleosomes with ADPr could facilitate transcription (see below). Previous studies have shown that cell lines do not entirely recapitulate the physiological responses to diverse stimuli as their primary culture counterparts, and these responses may differ significantly between transformed and primary cells (Ha et al. 2002; Berghaus et al. 2010). Indeed, previous studies from our lab have shown that the RAW 264.7 cell line responds differently to LPS stimulation versus LPS + IFN*γ* stimulation in terms of iNOS induction at the protein level (Ha et al. 2002), exemplifying that cell lines indeed may behave differently to primary cells. However, the use of the BV2 microglial cell line, which has been shown to behave comparably to native microglia (Ullrich et al. 2001) and responded comparably to native microglia in terms of inflammatory cytokine induction (Fig. 4E), was required in order to obtain enough chromatin material for the characterization of PARP1 enzymatic activity in facilitating inflammatory gene expression.

Recent evidence indicates a role for PARP2 and PARP3 in facilitating inflammatory gene expression in astrocytes (Phulwani and Kielian 2008), suggesting that other PARP family members besides PARP1 may be activated during CNS inflammation. However, as this study only reported the effects of PARP2 and PARP3 depletion, it is unclear whether these two enzymes were active in the conditions tested. Furthermore, the contribution of PARP2 and PARP3 to facilitating inflammatory gene expression is modest relative to that of PARP1 (Phulwani and Kielian 2008). Although pharmacological inhibitors, such as PJ34, are unable to discriminate which PARP family members could be activated in microglia, the greater abundance of PARP1 relative to other family members (Ame et al. 2004), our previous studies on PARP1-deficient glia (Ha et al. 2002; Ha 2004) and the enzymatic activity of recombinant PARP2 (Fig. 1F), and our data on LPS-stimulated BV2 cells indicate that PARP1 is in fact the major enzyme inhibited by PJ34.

While it is currently accepted that DNA damage underlies the enzymatic activation of PARP1 in neurons upon induction of CNS stress (Eliasson et al. 1997; Endres et al. 1998; Lee et al. 2000; Giovannelli et al. 2002), several mechanisms for PARP1 enzymatic activation in astrocytes and microglia have been proposed. For instance, the myelin- and membrane-derived cholesterol degradation products 7-ketocholestene (7-KC) (Diestel et al. 2003) and 15-hydroxycholestene (15-HC) (Farez et al. 2009) induce PARP1 enzymatic activity via DNA damage or a Toll-like receptor 2 (TLR2)-dependent signaling pathway, respectively. More specifically, recent evidence shows that distinct cellular signals including NGF (Cohen-Armon et al. 2007), LPS (Martinez-Zamudio and Ha 2012) and 15-HC (Farez et al. 2009) lead to PARP1 enzymatic activation through an ERK-dependent pathway. Results from our laboratory indicate that an ERK-dependent signal transduction pathway might also be involved in the enzymatic activation of PARP1 in stimulated microglia (R. I. Martinez-Zamudio and H. C. Ha, manuscript in preparation). The mechanism underlying this event remains to be determined, as we failed to detect activation of PARP1 by ERK through protein-protein interactions, both in vitro and in living cells (Martinez-Zamudio and Ha, manuscript in preparation). Putative ERK targets such as histones (Vicent et al. 2006), chromatin resident kinases (Ju et al. 2004; Vicent et al. 2006) and/or chromatin modifiers (Petesch and Lis 2012) may act to activate PARP1 in a gene promoter-specific manner in microglia by the modification of promoter chromatin structure. Supporting this notion, a recent study has demonstrated a sequential mechanism leading to the promoter-specific activation of PARP1. In this study, heat shock induces the acetylation of histone H2AK5 by the Tip60 complex at the *HSP70* locus. This modification results in the activation of promoter-bound PARP, which then promotes nucleosome displacement and increases *HSP70* transcription (Petesch and Lis 2012). Whether a similar mechanism operates downstream the ERK pathway in activated microglia remains to be investigated and will require the comprehensive identification of ERK-associated proteins in the chromatin fraction.

PARP1 plays an important role in mediating NF-*κ*B-dependent gene transcription occurs upon induction of inflammatory stresses (Ullrich et al. 2001; Ha 2004; Nakajima et al. 2004). For instance, an interaction between PARP1 and p65 is correlated with *Cd11a* expression in experimental models of ischemia-reperfusion (Ullrich et al. 2001) and infection (Nakajima et al. 2004). In addition, defective transcription factor activation and associated inflammatory gene expression are observed in PARP1-deficient glia (Ha 2004). The evidence from nucleosome remodeling assays and ChIP experiments presented here extends on these previous findings by providing insight into the molecular mechanism by which PARP1 enzymatic activity facilitates *Il1β* and *Tnf* expression in LPS-stimulated microglia.

Our nucleosome remodeling assays showed that PARP1 associates with both the 601 and *Il1β* nucleosomes and promotes their disassembly in a manner dependent on NAD^+^ concentration. Although both histones and PARP1 are modified under these conditions, subsequent analyses using purified pADPr-PARP1 demonstrated that histone ADP-ribosylation is responsible for the alteration in nucleosome structure. In contrast, extensively auto-modified PARP1 was unable to associate with nucleosomes and therefore unable to modify their structure. In addition, DNAse I hypersensitivity experiments showed that histone ADP-ribosylation increased the accessibility of nucleosomal DNA, even at NAD^+^ concentrations that did not promote nucleosome disassembly. Therefore, depending on the available NAD^+^ concentration, histone ADP-ribosylation may alter nucleosome structure ranging from discrete increases in accessibility to full nucleosome displacement. These results are in general agreement with seminal electron microscope data provided by the Poirier laboratory (Poirier et al. 1982), which showed that ADP-ribosylation promoted the relaxation of chromatin fibers through the ADP-ribosylation of the linker histone H1. Our results build on these previous findings by providing a function for core histone ADP-ribosylation in regulating local nucleosome structure. However, it remains technically challenging to determine the temporal order of PARP1 and nucleosome ADP-ribosylation in vitro. Determining the kinetics these processes, as well as the identification of target amino acid sites in core histones, will further define their specific functions in facilitating transcription and other chromatin-mediated processes.

Consistent with the in vitro assays, ChIP analyses revealed a constitutive association of PARP1 with the nucleosomes at the *Il1β* and *Tnf* promoters in resting microglia. Upon stimulation with LPS, a PARP1 enzymatic activity-dependent ADP-ribosylation at these promoters correlated with enhanced NF-*κ*B binding. In addition, the repressive effect of a PARP inhibitor on NF-*κ*B recruitment at the *Il1β* and *Tnf* promoters is paralleled with a dramatic reduction in their LPS stimulation-induced exp-ression. The mechanisms determining PARP1 positioning at these promoters remain to be established. However, the presence of nucleosomes at these promoters (Ramirez-Carrozzi et al. 2009) as well as the previously described nucleosome-binding properties of PARP1 (Kim et al. 2004; Martinez-Zamudio and Ha 2012) may in part explain this distribution. Whether PARP1 carries out additional functions at the *Il1β* and *Tnf* promoters remains unknown. It is possible that other mechanisms, such as pADPr-dependent recruitment of chromatin and transcriptional regulators (Petesch and Lis 2008; Zobeck et al. 2010), PARP1 interactions with NF-*κ*B (Ullrich et al. 2001; Hassa et al. 2003) and phosphorylation of NF-*κ*B (Ha 2004) are equally important for facilitating *Il1β* and *Tnf* expression in microglia. Nonetheless, the DNase I hypersensitivity and nucleosome remodeling assays presented here indicate that histone ADP-ribosylation is responsible for increasing accessibility of nucleosomal DNA, therefore increasing the probability of more stable NF-*κ*B binding to its cognate sites at the *Il1β* and *Tnf* promoters.

The resolution of our biotinylated-NAD^+^ ChIP assay prevented us from conclusively identify whether histone ADP-ribosylation or pADPr-PARP1 was present at the *Il1β* and *Tnf* promoters in stimulated microglia. However, the evidence from our in vitro studies prompts us to suggest that histone ADP-ribosylation likely enhanced recruitment of p65 at these promoters. It must also be considered that the resolution of ChIP followed by conventional PCR does not conclusively determine whether nucleosome displacement or slight alterations to nucleosome structure represent the main mechanism by which histone ADP-ribosylation facilitates p65 recruitment. Indeed, only a small fraction of the cell population may undergo nucleosome displacement at these promoters, which would obscure its detection in a population analysis such as ChIP. Nevertheless, the evidence presented here indicates that moderate alterations to the promoter chromatin structure may be sufficient for enhancing NF-*κ*B recruitment at the *Il1β* and *Tnf* promoters.

**Figure 5:**
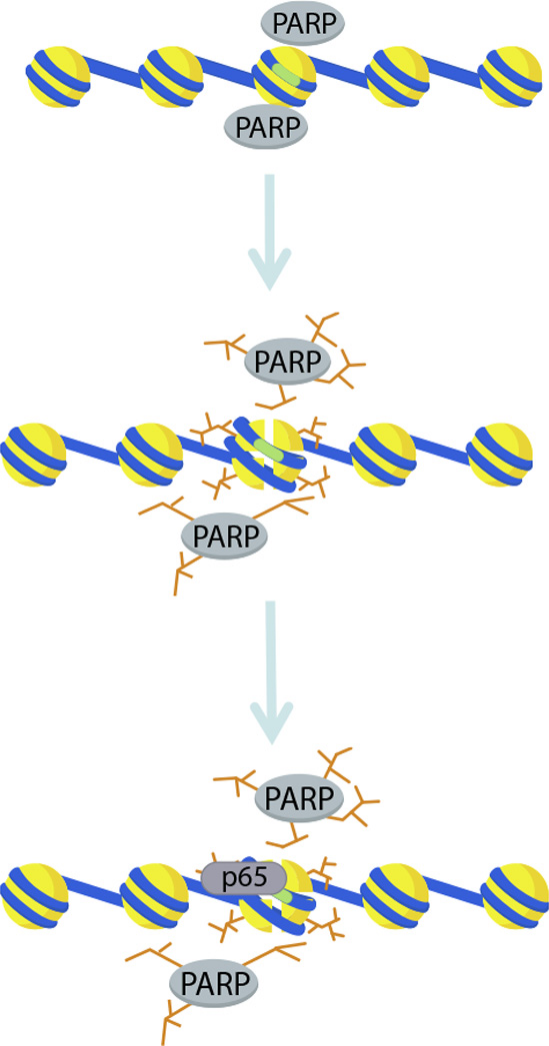
Hypothetical model of the epigenetic regulation of inflammatory cytokine expression by PARP1 in microglia. Top. In resting microglia, PARP1 is constitutively associated with the *Il1β* and *Tnf* promoter nucleosome(s) containing the NF-*κ*B-binding site (green highlight in center nucleosome). Middle. Upon stimulation, PARP1 is enzymatically activated and modifies itself and histones with ADPr. Bottom. Destabilization of histone-DNA interactions by ADP-ribosylation renders the *κ*B site accessible for p65 binding.

In summary, the data presented in this report favor a model in which nucleosome-bound PARP1 at the promoters of inflammatory cytokines is poised to facilitate their expression by remodeling nucleosome structure through histone ADP-ribosylation in response to environmental signals (Fig. 5). The increased accessibility of the promoter DNA enhances NF-*κ*B recruitment at these promoters and the subsequent transcription of these genes. These findings increase our understanding of the mechanistic role of PARP1 enzymatic activity during CNS inflammation. The identification of additional inflammatory genes regulated by PARP1-dependent nucleosome remodeling will provide an opportunity for the design of specific therapeutic strategies for inflammatory conditions of the CNS.

## Supporting Information

**Figure S1. Related to Figure 3.** Nucleosomes reconstituted with the 601 sequence were incubated with PARP1 or 200 *μ*mol/L NAD^+^ for 20 min at room temperature. Subsequently, reactions were incubated with DNase I and analyzed in an 8% denaturing polyacrylamide gel followed by autoradiography.
