# Supplementary figures and images for: PARP1 enhances inflammatory cytokine expression by alteration of promoter chromatin structure in microglia
Source: Brain Behav. 2014 Jun 9;4(4):552–65. doi: 10.1002/brb3.239 (PMC4128037; doi:10.1002/brb3.239)

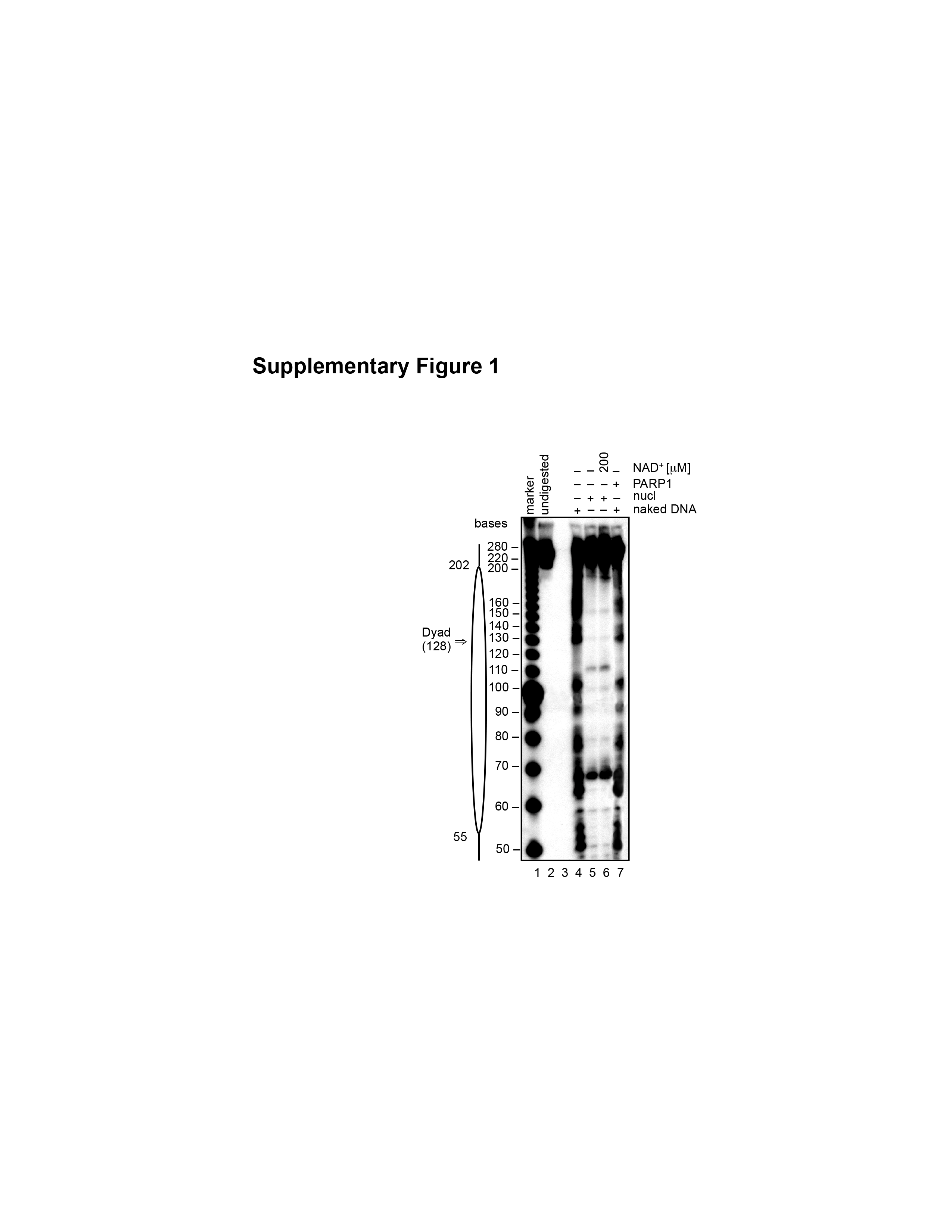

Supplement: Supplementary file 1 — Figure S1. Related to Figure 3. Nucleosomes reconstituted with the 601 sequence were incubated with PARP1 or 200 μmol/L NAD+ for 20 min at room temperature. Subsequently, reactions were incubated with DNase I and analyzed in an 8% denaturing polyacrylamide gel followed by autoradiography. [file brb30004-0552-SD1.png]
